# Thiol/Disulfide System Plays a Crucial Role in Redox Protection in the Acidophilic Iron-Oxidizing Bacterium *Leptospirillum ferriphilum*

**DOI:** 10.1371/journal.pone.0044576

**Published:** 2012-09-06

**Authors:** Javiera Norambuena, Rodrigo Flores, Juan P. Cárdenas, Raquel Quatrini, Renato Chávez, Gloria Levicán

**Affiliations:** 1 Laboratorio de Microbiología Básica y Aplicada, Departamento de Biología, Facultad de Química y Biología, Universidad de Santiago de Chile, Santiago, Chile; 2 Laboratorio de Ecofisiología Microbiana, Fundación Ciencia y Vida, Santiago, Chile; Ben-Gurion University of the Negev, United States of America

## Abstract

Thiol/disulfide systems are involved in the maintenance of the redox status of proteins and other molecules that contain thiol/disulfide groups. *Leptospirillum ferriphilum* DSM14647, an acidophilic bacterium that uses Fe^2+^ as electron donor, and withstands very high concentrations of iron and other redox active metals, is a good model to study how acidophiles preserve the thiol/disulfide balance. We studied the composition of thiol/disulfide systems and their role in the oxidative stress response in this extremophile bacterium. Bioinformatic analysis using genomic data and enzymatic assays using protein extracts from cells grown under oxidative stress revealed that the major thiol/disulfide system from *L. ferriphilum* are a cytoplasmic thioredoxin system (composed by thioredoxins Trx and thioredoxin reductase TR), periplasmic thiol oxidation system (DsbA/DsbB) and a *c*-type cytochrome maturation system (DsbD/DsbE). Upon exposure of *L. ferriphilum* to reactive oxygen species (ROS)-generating compounds, transcriptional activation of the genes encoding Trxs and the TR enzyme, which results in an increase of the corresponding activity, was observed. Altogether these data suggest that the thioredoxin-based thiol/disulfide system plays an important role in redox protection of *L. ferriphilum* favoring the survival of this microorganism under extreme environmental oxidative conditions.

## Introduction

Proteins of the bacterial periplasm and extracellular space often rely on disulfide bonds to support their correct folding and maintain their structural stability under oxidizing conditions. In contrast, intracellular proteins are contained within the reducing environment of the cytosol. Here, cysteine residues are reduced and often are involved in binding of substrate, coenzymes, or metal cofactors. They are present in the active site of enzymes participating directly in the catalyzed reaction. Moreover, cysteine residues are also involved in redox reactions, where the electrons transfer proceeds via thiol-disulfide exchange reactions [1]. Unlike periplasmic proteins, the activity of cytosolic enzymes depends on preserving the reduced state of the involved cysteine residues.

In the cytoplasm, one of the thiol/disulfide transition systems is represented by the small monomeric protein, thioredoxin (Trx) and by the NADPH–dependent flavoenzyme, thioredoxin reductase (TR). Cytoplasmic Trx possesses a conserved motif CXXC at the active site, where cysteines are responsible for reducing target proteins [2]. In the periplasm, the DsbA/DsbB system is responsible for the oxidation of thiol groups and the subsequent formation of disulfide bonds to fold proteins while the DsbC/DsbD system is responsible for the isomerization and shuffling of disulfide bonds and protein refolding [3]-[5]. Electrons from NADPH are provided to DsbD by the cytoplasmic thioredoxin system [5]. In *E. coli*, DsbD is also involved in the biogenesis of *c*-type cytochromes which play a role in reducing DsbE (CcmG), which in turn is responsible for maintaining reduced thiols present in apocytochromes for the subsequent heme insertion [3], [5].

In *E. coli* and other neutrophiles, another important thiol-disulfide exchange system exists. This system depends on glutathione (GSH), a tripeptide (Glu-Cys-Gly) that represents the main cytoplasmic cellular redox buffer [6]. The glutathione system is formed by glutaredoxins (Grx), the glutathione transferase (GST), the gluthatione reductase (GR) and NADPH. Glutaredoxins are GSH-disulfide oxidoreductases reported to catalyze a variety of GSH-dependent thiol-disulfide exchange reactions including protein glutathionylation and deglutathionylation. In turn GST catalyzes the formation of GSH conjugates and the reduction of hydroperoxides. All these functions involve the oxidation of the thiol group of GSH, primarily to form glutathione disulfide (GSSG). The GSH/GSSG ratio is maintained by the glutathione reductase (GR), a homodimeric flavoprotein that uses NADPH to reduce one GSSG molecule to two GSHs [5], [7]. Gram positive bacteria, and exceptionally some Gram negative bacteria as *H. pylori*, lack the enzymes to generate glutathione and other thiol reductants such as glutaredoxin. In these microorganisms thioredoxin, but not glutathione, plays a crucial role in the maintenance of the thiol/disulfide balance in the cell [5], [8], [9].

*Leptospirillum ferriphilum*, an acidophilic bacterium that uses Fe^2+^ as electron donor, and withstands very high concentrations of this and other redox active metals, is a remarkable model to study how acidophiles confront oxidative stress and how they preserve the thiol/disulfide balance. This microorganism is important for bioleaching of sulfide ores and the recovery of industrially important metals. Bioleaching processes are carried out in extremely acidic conditions (∼pH 1) where extraordinarily high concentrations of soluble iron and heavy metals are present [10]. These conditions are harmful for the vast majority of microorganisms, as they induce oxidative stress through the generation of reactive oxygen species (ROS) leading to damage of biomolecules and cell death [11]. Additionally, as iron is an energy-poor substrate, ferrous-oxidizing bacteria maintain the membrane potential through the oxidation of iron at very high rates [12], also favoring the generation of ROS. Although some knowledge has been gained in recent years regarding the oxidative stress response in a few acidophilic iron-oxidizing bacteria and archaea [13], [14], the general strategies used by these organisms to face ROS challenges are still inadequately understood.

In this work the composition and activity of the thiol/disulfide system of *L. ferriphilum* DSM 14647 is analyzed. We provide evidence that in this acidophile the thioredoxin system, but not glutathione, plays a pivotal role in defense against different oxidative conditions.

## Materials and Methods

### Bacterial strains and Growth Conditions

*L. ferriphilum* DSM 14647 was grown in 882 medium in accordance with German Collection of Microorganisms and Cell Cultures (DSMZ) recommendations. *E. coli* ATTC 4468 and *Bacillus subtilis* HB 7038 were grown in Luria-Bertani (LB) medium. All bacteria were grown aerobically at 37°C with constant stirring. *Helicobacter pylori* ATCC 700392 was grown according to Cerda et al. [15].

### Bioinformatic Analysis

Protein sequences related to the Trx and GSH systems obtained from the KEGG database (http://www.genome.jp/kegg/pathway.html) were used as queries. Genome shotgun sequences for *Leptospirillum* Group II '5-way CG', *L. rubarum* and *L. ferrodiazotrophum* publically available at National Center for Biotechnology Information (NCBI) database (http://www.ncbi.nlm.nih.gov/bioproject/29591) were searched using tBLASTn [16] with default parameters. When a prospective candidate gene was identified, its predicted amino acid sequence was used to formulate a BLASTP search against the NCBI non-redundant database. Candidate genes and their corresponding translated proteins were further characterized using several bioinformatics tools. Primary structure similarity relationships were determined using ClustalW 1.8 [17]. Structural motif predictions were performed using Prosite [18]. Cellular location was determined using PSORT [19], SignalP [20], TMPred [21], and Pred TMBB [22]. Peptide domain predictions were done using ProDom [23]. Theoretical isoelectric point (*pI*) and molecular weight (MW) was computed using Compute pI/MW [24]. Sequences used in ClustalW analysis and phylogram tree construction were: DsbD (ZP_02809776.1), CcdA (CAP07666.1), DsbC (NP_311792.1), DsbE (NP_311111.1), DsbG (AAC45785.1), TrxA (AAC76786.2), TrxC (NP_289141.1) and two peroxiredoxins (ACT30056.1 and ACA76862.1) from *E. coli*; DsbD (NP_391228.1), TrxA (P14949) from *B. subtilis*; Trx1 (AAA88939.1) from *A. ferrooxidans*; TrxA (YP_210347.1) and TrxG (YP_210941) from *Bacteroides fragilis*; DsbA (ZP_07393500) from *Shigella flexneri*; DsbA (ZP_07393500) from *Shewanella baltica*; DsbA (YP_003085970) from *Dyadobacter fermentans* and TrxA (NP_247280.1) from *Methanocaldococcus jannaschii*.

### Cell Extract Preparation

Overnight cultures of *B. subtilis, H. pylori* and *E. coli* were transferred to fresh LB medium and grown to OD_600_ ∼0.5. Cultures were incubated aerobically at 37°C for 1 h with 4 mM diamide. Cells were harvested by centrifugation at 8,000×g for 3 min, washed twice with 50 mM HEPES buffer pH 8.1, suspended in lysis buffer (20 mM HEPES pH 8.1, 2 mM EDTA, 200 mM KCl, 0.1% Triton X-100, 2 mM PMSF, 0.2 mg/ml lysozyme and incubated for 30 min at 30°C. Cells were disrupted by sonication and centrifuged twice at 13,000 x g for 20 min.

*L. ferriphilum* was grown until late exponential phase and cells were harvested by centrifugation at 8,000 x g for 15 min, washed once with acid water (pH 1.4) and twice with 10 mM sodium citrate pH 6.0. The washed cells were suspended in 882 medium and incubated with 1 mM H_2_O_2_, 4 mM diamide or 150, 250, 260 mM Fe^3+^ [Fe_2_(SO_4_)_ 3_] for the indicated time. To avoid H_2_O_2_ reacting with ferrous iron and the subsequent generation of OH^.^ (Fenton reaction), the medium was not amended with FeSO_4_×7H_2_O in the corresponding treatment. After exposure to oxidative agents, cells were harvested and washed twice with 10 mM sodium citrate pH 6.0. The bacterial pellet was suspended in lysis buffer (30 mM HEPES, pH 8.0, 150 mM NaCl, 1 mM DTT) and incubated for 30 min. Cells were disrupted by cycles of freezing at −78°C and thawing by sonication. Extracts were centrifuged at 20,000 ×*g* for 30 min. Supernatants were ultracentrifuged at 150,000 ×*g* for 90 min. When required, the cellular extract was dialyzed against lysis buffer containing 50% glycerol. Aliquots were stored at −80°C. Protein concentration was determined as described by Bradford [25].

### Glutathione Reductase (GR) Assay

GR was assayed as described [26]. Briefly, the reaction mixture (500 µl) contained 1 mM oxidized glutathione, 100 mM Tris-HCl buffer, 0.2 mM NADPH, 2 mM EDTA and 0.1 mg/ml BSA. Reactions were started by adding 50 µg of cell extract and activity was monitored at 340 nm. Blank contained all the components except the protein extract. One unit of GR activity was defined as 1 µmol of oxidized NAPDH by µg of protein per min. All measurements were carried out at room temperature using an UVmini-1240 spectrophotometer (Shimadzu).

### Insulin Reduction Assay

Trx activity was determined according to Arnér and Holmgren [27], with minor modifications. Briefly, reaction mixture (500 µl) contained TE buffer (50 mM Tris-HCl, 1 mM EDTA, pH 7.5), 0.16 mM insulin (Sigma) and cell extract (25 µg of total protein extract from *L. ferriphilum* or *B. subtilis*, or 50 µg from *E. coli*). The reaction was started by adding of 0.33 mM DTT. The absorbance at 650 nm was monitored at room temperature.

### DTNB Reduction Assay

*L. ferriphilum* TR activity was determined as described [28]. Briefly, the reaction mixture (550 µl) contained 100 mM phosphate buffer pH 7.0, 2 mM EDTA, 0.1 mg/ml BSA, 5 mM DTNB and 300 µM NADPH. Reactions were started by adding 25 µg of cell protein extract. The increase in absorbance at 412 nm due to production of 3-carboxy-4-nitrobenzenethiol (NBT), was monitored for 3 min. Activity was calculated by using a molar extinction coefficient of 13,600 M^−1^cm^−1^ [29].

### RNA Isolation and cDNA Synthesis

*L. ferriphilum* was grown until late exponential phase. Cells were harvested by centrifugation at 8,000 ×g for 15 min and washed once with acid water and twice with 10 mM sodium citrate pH 6.0. Washed cells were suspended in 882 medium and incubated with 1 mM H_2_O_2_, 4 mM diamide or 260 mM Fe^3+^ for the time indicated. Cells were collected by centrifugation at 8,000×g for 5 min, washed twice with 10 mM sodium citrate pH 6.0. RNA was isolated using the Trizol (Invitrogen). DNA was removed by DNase I treatment (Invitrogen) according to manufacturer’s instructions. cDNA synthesis was carried out with M-MuLV reverse transcriptase. Reaction mixture contained 0.25 mM of each primer, 0.5 mM dNTPs, 200 U M-MuLV enzyme, 2 µl M-MuLV buffer and 500 U RNase inhibitor. Synthesis was carried out at 42°C for 1 h and the enzyme was inactivated at 95°C for 15 min. cDNA was stored at −20°C until further use.

### Quantitative PCR

Primers for all RT and PCR reactions were designed within conserved regions of each gene (Table 1). For this, alignments of nucleotide sequences from *Leptospirillum* Group II '5-way CG', *L. rubarum*, and *L. ferrodiazotrophum* were carried out using ClustalW. *RrsB* gene encoding for 16S rRNA was selected as housekeeping gene [30] for normalizing *trx* gene expression.

**Table 1:** Primers used in this work.

Gene	Primer	Sequence	Fragmentsize (bp)
*trx 1*	P1-F	GGAAAAGAACTGGCTCAACG	111
	P1-R	AGGAGGTGAATGGCCCGCTGG	
*trx 3*	P3-F	ATCATCGGGATTCATACTC	194
	P3-R	CGATGGAATGGTATTGGAG	
*trx 4*	P4-F	TGGTGKGTTCCCTGCC	96
	P4-R	KGTCCATCGCCACGCTS	
*trx 5*	P5-F	GGATTACAGGGGAAAAGTC	127
	P5-R	GGCGCTACTTTGTTGGC	
*trx 6*	P6-F	CTGGCCCGAAAGTACCACGG	87
	P6-R	CATCGCCTCCATCTGTCGG	
*trx 7*	P7-F	CTGGGCAACCTGGTGTGG	91
	P7-R	TCCCAGAATGACSACCCG	
*trx 8*	P8-F	GCCCTSATCGATCCGG	60
	P8-R	CKCCCTCTTTCCTTCYCC	
*trx 11*	P11-F	CAYTGGTGCCATGTCATGG	230
	P11-R	TGAGCHGGGAAATAGGTTCC	
*trx 13*	Ptr-F	TTGCGATTCGGGACCC	133
	Ptr-R	CCGACGCTCCACTGGC	
*rrsB*	16S-F	ACGGGTGAGTAAGACATGGG	112
	16S-R	GGCCTCCCTTTCCCCG	

Quantitative PCR reaction mixture contained 12.5 µl of 2X SensiMix SYBR Kit (Quantace), 2 µl cDNA, 0.5 mM of each primer and 8.5 µl H_2_O. The thermal cycling conditions were an initial denaturation at 95°C for 5 min, followed by 35 cycles of denaturation (95°C for 10 sec), annealing (for 15 sec) and extension (72°C for 10 sec), followed by fluorescence measurement and a final melt curve (50–99°C). The annealing temperature was different for each set of primers: *trxB* (58°C), *trx1* and *trx6* (61°C) and *rrsB* (59°C). Three independent trials were averaged in all cases. A reaction mixture without cDNA was run as control for detecting DNA contamination. All this reactions were performed in Rotor-Gene 6000 (Corbett Research) thermocycler.

### Statistical Analysis

Statistical analysis was performed by using ANOVA followed by Turkey’s test using GraphPad Prism 5. The differences were considered significant at p<0.05.

## Results

### *In silico* Identification of Thiol-protective Systems Genes in *Leptospirillum* Genomes

To gain insight into the thiol/disulfide balancing mechanisms used by the Leptospirilli the whole genome shotgun sequence of *Leptospirillum* “5-way CG” (available at National Center for Biotechnology Information NCBI database) was analyzed. No candidate genes related to a glutathione system were found; no orthologues for glutathione reductase, glutathione-S transferase, glutathione hydrolase and glutaredoxins were found in this genome. In accordance, neither of the key enzymes of the glutathione tripeptide synthesis pathway, γ-glutamylcysteine synthetase or glutathione synthetase, were predicted. Similar results were found in *L. rubarum* and *L. ferrodiazotrophum* genomes. In contrast, a number of thioredoxin candidate genes related to thioredoxin family were found. Putative role of the predicted thioredoxins was inferred by employing multiple sequence alignment tools (ClustalW) and phylogram trees including well known thioredoxins (Trx) and thioredoxin reductase (TR) protein sequences.

The analysis revealed that *Leptospirillum* “5-way CG” possesses genes encoding four proteins that belong to the thioredoxin system (Table 2, Figure S1). Three of these proteins are predicted thioredoxin (Trx1, Trx2 and Trx6) and one is a putative thioredoxin reductase (TR). Experiments were conducted to amplify these *trx* genes in *L. ferriphilum*. Products for *trx1* and *trx6* genes, and TR encoding gene (*trxB*) were obtained (not shown). Thioredoxin 2 (Trx2) and 6 (Trx6) were related to bacterial TrxA and possess the characteristic WCGPC motif [31]. On the other hand, thioredoxin 1 (Trx1) was more related to the archaeal Trx (Figure S1). Gene context analyses showed that *trx1* is immediately adjacent to genes *qcrB* and *qcrA* known to be involved in the biogenesis of cytochrome *bc*_1_ in several microorganisms, suggesting that Trx1 from Leptospirilli is probably involved in the maturation of this redox complex (data not shown). The predicted TR from *Leptospirillum* “5-way CG” conserves the typical CATC motif described for this enzyme [32].

**Table 2:** Bioinformatic characterization of predicted Trx.

Protein	Possible function	Localization	pI/MW**	Accession
Trx 1*	Thioredoxin (TrxA)	Undetermined	9.5/9.5	EDZ38669.1
Trx 2	Thioredoxin (TrxA)	Cytoplasm	5.4/12.1	EAY55688.1
Trx 3*	Cytochrome *c* maturation (DsbE)	Undetermined	8.7/38.9	EDZ39861.1
Trx 4*	Cytochrome *c* maturation (DsbE)	Cytoplasmic membrane	6.4/23.6	EDZ38907.1
Trx 5*	Cytochrome *c* maturation (DsbE)	Cytoplasmic membrane	9.8/22.5	EDZ40037.1
Trx 6*	Thioredoxin (TrxA)	Cytoplasm	7.8/12.4	EDZ39468.1
Trx 7*	Cytochrome *c* maturation (DsbE)	Cytoplasmic membrane	9.5/19.1	EDZ40036.1
Trx 8*	Isomerization of disulde bonds DsbD	Undetermined	5.4/54.0	EDZ39661.1
Trx 9	Thiol oxidation (DsbA)	Undetermined	6.2/24.7	EDZ39344.1
Trx 10	Peroxiredoxin (Prx)	Cytoplasm	6.8/17.0	EDZ38626.1
Trx 11*	Isomerization of disulde bonds DsbD	Undetermined	5.7/78.4	EDZ40070.1
Trx 12	DsbD-like	Undetermined	6.7/28.0	EDZ38693.1
Trx 13*	Thioredoxin reductase (TR)	Cytoplasm	6.3/33.5	EDZ40073.1

Amino acid sequences from *Leptospirillum* “5 way CG” were obtained from NCBI data base.

* Encoding genes detected by PCR method in *L. ferriphilum* DSM14647.

** pI/MW was calculated for the precursor protein.

The remaining thioredoxin family proteins identified in the *Leptospirillum* “5-way CG” whole genome shotgun sequence were Dsb proteins and peroxiredoxins. The DsbA/DsbB proteins forms part of the thiol oxidation system involved in the proper folding of secreted and periplasmic proteins [5], where DsbA is a known member of the thioredoxin family [3]. Both DsbA (Trx9) and DsbB candidate protein encoding genes were found in *Leptospirillum* “5-way CG”. Predicted DsbA lacks the second cysteine in the active site and has low similarity with DsbA from *E. coli* (41%). However, a relative conservation of the *dsbB* gene (45% similar to the *E. coli* ortholog) suggest that DsbA and DsbB proteins could play a role in thiol oxidation and folding of exported proteins in *Leptospirillum spp*.

In *E. coli*, the isomerization system is composed by DsbC/DsbD proteins. DsbD (59 kDa) exhibits 3 characteristic domains: an immunoglobulin like domain (DsbDα), a hydrophobic domain with 8 transmembrane helixes (DsbDβ) and a thioredoxin domain (DsbDγ) [3]. *Leptospirillum* “5-way CG” genome encodes one candidate *dsbD* gene (*trx12*), however its predicted product is smaller than DsbD from *E. coli* and lacks the DsbDα and DsbDβ domains. In addition, the accompanying *dsbC* gene candidate is missing in all three sequenced Leptospirilli reported so far, suggesting that the DsbC/DsbD system is not present as such in this species group. As discussed below, the role of the predicted DsbD-like thioredoxin could be related to *c*-type cytochrome maturation.

It has been reported that Trx-like proteins are involved in the biogenesis of cytochrome *c*. In *E. coli* DsbD/DsbE proteins keep the apocytochrome reduced prior to the insertion of the heme group. DsbE directly reduces the CXXCH motif in the apocytochromes and a protein similar to DsbD regenerates the reduced state of DsbE in a NADPH-dependent reaction [33]. According to our bioinformatic analysis, *Leptospirillum* “5-way CG” possesses two additional genes coding for DsbD related proteins (*trx8* and *trx11*) and at least three DsbE encoding orthologues (*trx4*, *trx5* and *trx7*). Although more distant, Trx3 also appears to be related to DsbE. PCR assays carried out to detect genes that encode for these proteins in *L. ferriphilum* DSM 14647 revealed that all of them are well conserved (not shown). All putative DsbE proteins have a similar size to DsbE from *E. coli* [34] and are predicted to be anchored to the cytoplasmic membrane, as in other microorganisms [3], [5], [35]. Interestingly and according to their genetic context, Trx5- and Trx7-encoding genes could be forming a transcriptional unit with genes related to system II of cytochrome *c* biogenesis, as those encoding *CcsA*, *CcdA*, *ResA* and *ResB* proteins (not shown). Such functional association strongly suggests that *trx5* and *trx7* genes encode functional DsbEs related to maturation of *c*-type cytochromes. Implications of this apparent redundancy of DsbE in *Leptospirillum spp*. will be discussed below.

Finally, Trx10 is a similar to peroxiredoxin. In this context, it is important to point out that the assembly of different trx fold domains has been used many times during evolution to build new proteins that perform a large number of catalytic functions. Peroxiredoxins (Prxs) involved in (in)organic peroxides reduction [36] belong to this new and multi-domain proteins. Thus, although Trx10 is not a thioredoxin itself, it is indeed evolutionarily related to this family of proteins.

Altogether, these results suggest that in *Leptospirillum spp.* the thioredoxin system, but not glutathione, plays a fundamental role in the control of the cellular thiol/disulfide redox balance. Furthermore, in this genus there are a number of proteins belonging to thioredoxin family that are predicted to be involved in thiol oxidation and *c*-type cytochrome maturation in the periplasm.

### Glutathione System

**Figure 1:**
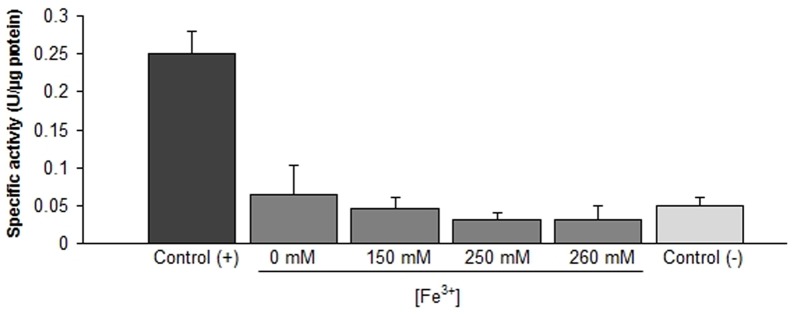
Glutathione reductase activity. The protein crude extract from *L. ferriphilum* (gray) were obtained after 1 hour of exposure to 0, 150, 250, or 260 mM Fe^3+^. *E. coli* (black) and *H. pylori* (light gray) were used as positive and negative controls, respectively. Bars represent µmol of oxidized NADPH by µgram of protein per min during 15 min of incubation (initial velocity). The average was calculated from three independent trials (lines on top of bars represent values range).

A recent report has suggested that glutathione regeneration appears to be involved in As(III) tolerance in *L. ferriphilum* [37]. This sharply contrasts the bioinformatic evidence generated in this study for the sequenced Leptospirilli, all of which lack of known glutathione related genes. In order to resolve this controversy, glutathione reductase (GR) activity was assayed on cellular extracts of *L. ferriphilum* DSM 14647 grown under standard and oxidative stress conditions. Oxidative stress was induced for 1 hour using ferric iron in a range of 150–260 mM, as described in more detail in materials and methods. Cellular ROS overproduction under these conditions has been previously demonstrated [38]. Total cellular extracts from *Escherichia coli* and *Helicobacter pylori* were used as positive and negative control of GR activity, respectively [9]. In agreement with our bioinformatic results, no GR activity was detected in cellular extracts under both standard and oxidative stress conditions (Figure 1). These results support the idea that *Leptospirillum spp*. does not use glutathione as redox buffer.

### Thioredoxin System

To analyze the activity and involvement of the thioredoxin system in the oxidative stress response of the Leptospirilli we measured the activity of both Trx and TR in cellular extracts from *L. ferriphilum* exposed to different oxidative stress elicitors. Diamide, was used as positive control of oxidative stress because of its capacity to induce generalized disulfide stress [39].

### Thioredoxin Activity

**Figure 2:**
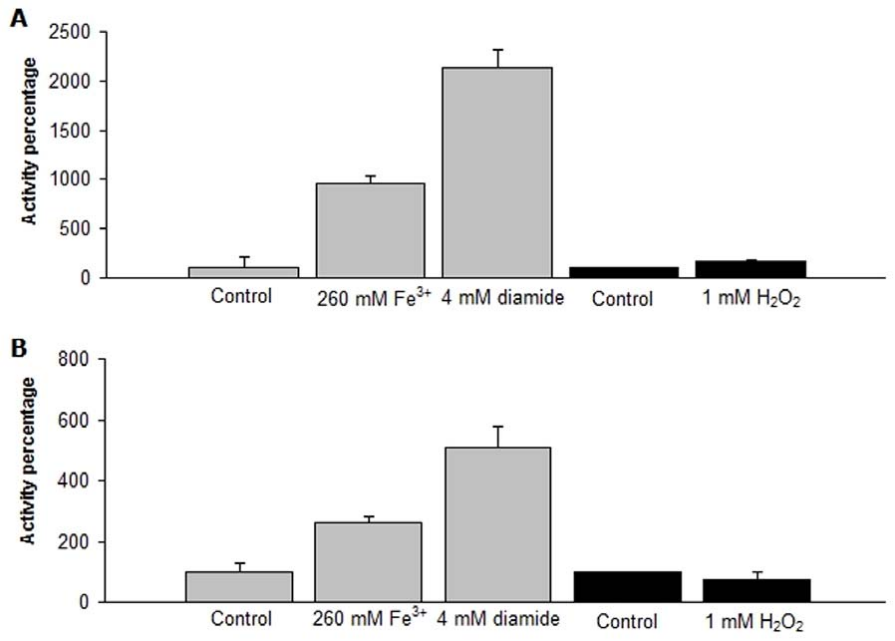
Thioredoxin activity. The reduction of the insulin alfa-chain was monitored at 650 nm in bacteria treated with 260 mM Fe^3+^, 4 mM diamide, or 1 mM H_2_O_2_, at 30 (A) or 60 minutes (B) of stress exposure. The reaction was performed as described in materials and methods after 15 min of incubation. Activity in the control reaction corresponds to 100%. Data represents the average of two independent trials (lines on top of bars indicate values range). The negative control (mixture without protein) did not reduced insulin alfa-chain at 15 min.

Trx activity was measured in whole cellular extract derived from cells exposed to 260 mM ferric iron, 4 mM diamide or 1 mM H_2_O_2_ for 30 or 60 min. As is shown in Figure 2, at 30 minutes the thioredoxin activity increased 9-and 20-fold in response to exposure to ferric iron or diamide, respectively. In addition, Trx activity increased in a dose-dependent way under exposure to different concentration of ferric iron (data not shown). By contrast, there was not significant increase of the activity at 30 min exposure to H_2_O_2_. At 60 min exposure, thioredoxin activity greatly decreased with ferric iron (4-fold) and diamide (5-fold) compared to 30 min, however the percentage of activity was maintained higher than the control (100%). These results clearly indicate that *L. ferriphilum* possesses functional thioredoxin(s) whose activity can be induced by exposure to oxidative stress.

It is important to note that data normalization of Fe^3+^ and H_2_O_2_ stress experiments relative to their corresponding controls (percentage of activity) precludes thioredoxin activity differences to be seen among control experiments. However, at 30 minutes the activity in the control reaction for the H_2_O_2_ treatment increased 25-fold with respect to the Fe^3+^ control reaction. As mentioned above, Fe^2+^ was included in the experiment in which cells were exposed to Fe^3+^, but not to H_2_O_2_. Since Fe^2+^ represents the only energy source for *L. ferriphilum*, its absence probably triggered a starvation state in this iron-oxidizing bacterium and a concomitant increase of Trx activity. This observation is in agreement with previous studies showing that the Trx system from *E. coli* is induced under nutrient deficiency [9]. This result suggests that *L. ferriphilum* starved cells (in H_2_O_2_ assays) are somehow facing oxidative stress.

**Figure 3:**
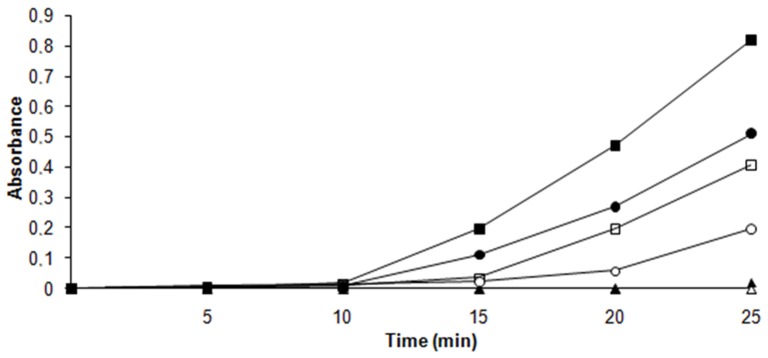
Comparison of thioredoxin activity between *L. ferriphilum* and neutrophilic bacteria. The reduction of the insulin alfa-chain was monitored at 650 nm for 25 minutes in *E. coli* (triangle), *B. subtilis* (circle) and *L. ferriphilum* (square) after 4 mM diamide exposure during 60 minutes (close figures). Controls are shown with open figures. The negative control (mixture without protein) did not reduced insulin at 30 min. This data represents 2 independent trials.

*B. subtilis* and *E. coli* are neutrophilic microorganisms described as possessing, respectively, thioredoxin or glutathione as the main thiol-reducing systems of the cell [5], [9]. Interestingly, comparison of the insulin reduction as an indicator of Trx activity between *L. ferriphilum* DSM 14647 and these neutrophilic bacteria (Figure 3) revealed that in presence of the disulfide stress elicitor diamide, both *L. ferriphilum* and *B. subtilis* respond similarly, with a steady increase in activity upon diamide exposure with respect to the control. In spite of this, *L. ferriphilum* showed higher activity than *B. subtilis* at all time points assayed in both control and disulfide stressed conditions. Instead, in *E. coli* an increase of activity was not observed. These data show that the Trx system from *L. ferriphilum* is highly active and strongly suggest that in *Leptospirillum* spp. thioredoxins represent a key mechanism to respond to the extreme oxidative conditions of bioleaching environments.

#### Thioredoxin reductase activity

**Figure 4:**
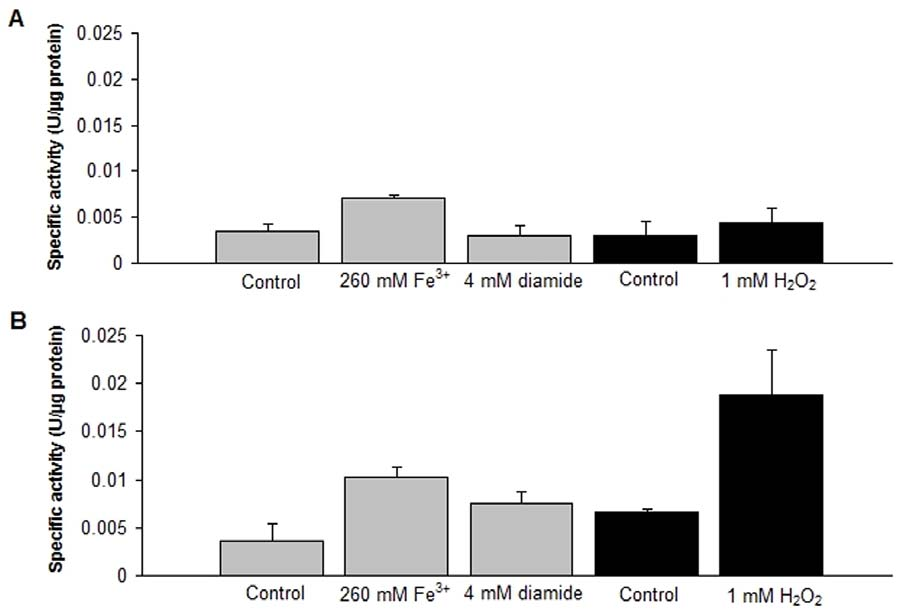
Thioredoxin reductase activity. The reduction of DTNB to TNB was monitored at 412 nm in bacteria treated with 260 mM Fe^3+^, 4 mM diamide, or 1 mM H_2_O_2_, at 30 (A) or 60 minutes (B) of stress exposure. The reaction was performed as described in materials and methods during 3 min of incubation (initial velocity). The data is normalized by the negative control (mixture without protein). This data represents the mean of 2 independent trials. Bars indicate values range.

TR activity was measured using the DTNB reduction method in whole cellular extracts derived from cells grown under oxidative stress conditions during 30 or 60 min. It should be noted that 0.6 µM auranofin, an extremely potent inhibitor of TR [40], inhibited the TR activity in the extracts derived from *L. ferriphilum* grown under standard conditions by about 70% after 10 min of incubation, showing that the vast majority of the measured DTNB reduction is specifically mediated by TR activity. As shown in Figure 4, after 30 minutes of oxidative stress elicited by 260 mM Fe^3+^, 4 mM diamide or 1 mM H_2_O_2_, TR activity exhibited a very modest increase with respect to the corresponding controls. Nevertheless, at 60 min of exposition of the cells to H_2_O_2_, TR activity showed a significant increase, nearly 3-fold greater than the control. The effect of the Fe^3+^ and diamide was again modest and comparable to the observed at 30 min. These data indicate that TR activity reaches a peak later than Trx activity, which peaked at 30 min. In addition, TR but not Trx activity, responded to H_2_O_2_ indicating that different stimuli could mediate regulation of each activity in a timely fashion. We propose that Trx provides an early response to confront oxidative stress, whereby redox balance is preserved and oxidized thioredoxins are accumulated. This leads to a decrease in Trx activity that could be restored to some extent with an increase in TR activity.

**Figure 5:**
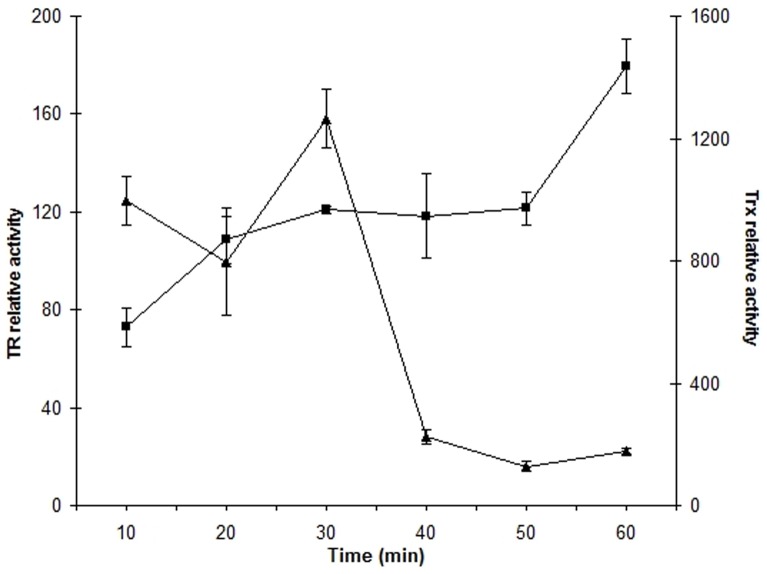
Time-course curve of Trx and TR relative activity. Activities of TR (square) and Trx (triangle) were measured every 10 minutes until 60 minutes after exposure to 260 mM Fe^3+^. This data represents the mean of 3 independent trials (bars indicate values range). All data are normalized by their respective controls.

To evaluate whether *L. ferriphilum* Trx and TR enzymes are activated coordinately in response to oxidative stress, we measured both activities in parallel during a 60 min time lapse in whole extracts from cells exposed to 260 mM Fe^3+^. As shown in Figure 5, Trx activity raised in time reaching a maximum at 30 min and then declined rapidly over time (4-fold in 10 min). Following a different scheme, TR activity increased steadily, reaching its maximum only after 60 min (1.5-fold).

The decrease in Trx activity observed during the first 20 min may indicate that cellular thioredoxins are inactivated by oxidation. Increase of TR activity could compensate the initial decrease of Trx activity, achieving a new maximum at 30 min. However, the sustained increase in TR activity (during 60 min period) appears to be insufficient to restore Trx activity to the values observed in the control condition after 30 min. This data clearly support the idea that in *L. ferriphilum* there is a closely related response of Trx and TR activity to cope with oxidative stress as proposed above.

### Expression of Genes Coding for Thioredoxin System

**Figure 6:**
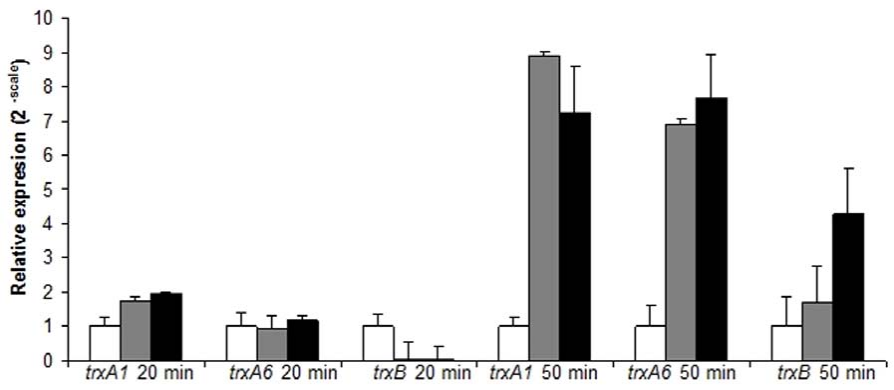
Relative expression of *trx* genes in oxidative stressed *L. ferriphilum*. Bacteria were treated with 260 mM Fe^3+^ (gray) or 4 mM diamide (Black) for 20 or 50 min. Cells grown under standard growth conditions were used as negative control (white). Data was normalized by 16S rRNA. Data represents the average of 3 independent trials (bars indicate values range).

Since ROS-mediated stress, results in increased activity of thioredoxin system of *L. ferriphilum,* the transcriptional response of this bacterium under oxidative stress treatment was assessed. We analyzed the expression profiles of *trx1* and *trx6* genes that code for cytoplasmic thioredoxins, and *trxB* gene that code for the TR enzyme from *L. ferriphilum* DSM 14647. Expression level of each gene was quantified 20 or 50 min after exposure to the oxidative stress generating compounds using real-time RT-PCR. As shown in Figure 6, *trx1* gene expression was up-regulated by Fe^3+^ and diamide treatment after 20 min. Similarly, *trx6* was slightly up-regulated under oxidative stress by diamide. In contrast, *trxB* gene was repressed in both Fe^3+^ and diamide stress conditions.

Interestingly, after 50 min of treatment with any of the oxidative stress elicitors, all three genes investigated, *trx1*, *trx6* and *trxB,* were significantly up-regulated. Expression profile of *trxB* gene is coincident with the higher activity of TR enzyme observed at 60 min shown above. However, in case of the *trx1* and *trx6* genes, the sustained increase expression at 20 and 50 min under oxidative stress were not coincident with Trx activity measured at 30 and 60 min. It should be mentioned that under stress Trx activity resulted to be conversely higher at 30 min than 60 min. Such difference is likely attributable to the oxidative damage suffered by thioredoxins during the oxidative stress condition.

## Discussion

In this study a bioinformatic and biochemical characterization of the thiol/disulfide system of the extreme acidophilic bacterium *L. ferriphilum* was undertaken. Unlike most eukaryotes and many other prokaryotes (mainly Gram-negative bacteria), members of the *Leptospirillum* genus lack the genes encoding enzymes to generate glutathione and other thiol reductants such as glutaredoxins. In agreement with this observation, glutathione reductase activity responsible for maintaining glutathione in its reduced state proved to be absent in *L. ferriphilum* whole cell extracts. Although this aspect should be investigated in more detail, our data certainly support the idea that a glutathione-based thiol/disulfide system is not present in *Leptospirillum spp*. In other organisms, the presence of alternative functional thiols as mycothiol [41], cysteine [42], coenzyme A [43], thiol-cobalamin [44] or bacillithiol [45] has been described. Further research will be required to determine whether other low molecular weight thiols are also synthesized in the Leptospirilli.

On the other hand, results presented herein indicate that the Leptospirilli, including *L. ferriphilum,* encode all key gene products of the thioredoxin-based thiol/disulfide system. These proteins could participate in many crucial cellular functions, including oxidative stress management, refolding of exported proteins and cytochrome *c*-type maturation. Results presented in this work are consistent with previous metaproteomic data from the Iron Mountain acid mine drainage biofilm community from which the genomic data analyzed herein was also derived [46], [47]. In that study *Leptospirillum* group II was found to be the dominant microorganism (48% of the metaproteome) and thioredoxins and disulfide isomerases a highly represented protein category (9% of the metaproteome) [48]. Our interpretation is that they are very important for preserving redox balance in this group of acidophiles when ROS generating conditions are present in the environment.

Evidence presented in this work, shows that exposure of *L. ferriphilum* to H_2_O_2_, in the absence of Fe^2+^ as energy source, triggers a response of the thioredoxin system apparently related with starvation. As deduced from the comparison of the controls treatments for H_2_O_2_ (without Fe^2+^) and Fe^3+^/diamide stress experiments, Fe^2+^ deficiency provoked the induction of Trx activity. Induction of the oxidative stress response under nutrient limitation has already been observed in *E. coli* at the onset of stationary phase [49]. Also in *Lactococcus lactis* an oxidative stress response was activated as an adaptation to isoleucine starvation [50]. Connection of this response with intracellular oxidative stress still awaits elucidation.

One unexpected finding of this work was the identification of four copies of *dsbE* gene. Such as mentioned above, DsbE is directly involved in maintenance of the reduced sate of the apocytochrome *c* prior insertion of heme [51], [52]. In a previous work we could establish that members of *Leptospirillum* genus possess a high number (n = 18–20) of genes e*n*coding *c*-type cytochromes [53]. This positive correlation tempted us to speculate that redundancy of *dsbE* genes could be a requirement to achieve high levels of DsbE proteins in order to satisfy the high demand of mature and functional cytochromes required for iron oxidation metabolism [12], [54], [55].

Despite a great deal of information on the kinetics of individual thioredoxin-dependent reactions, the kinetic regulation of the thioredoxin system as an integrated whole remains almost unknown. Recently, Pillay et al. [56], using a realistic computational model, found that decreases in the concentration of TR triggered decreases in the fluxes of all thioredoxin-dependent reactions, showing that the kinetic profiles for all reactions that yield oxidized thioredoxin can be affected. In a similar way, we detected that an initial increase in TR activity leads to an increase in Trx activity. However, while TR activity increased steadily during the first 60 min of exposure to oxidative stress, Trx activity decreased dramatically after 30 min period. Based on the data obtained herein, we interpret that in the struggle to maintain a reducing environment TR activity becomes overwhelmed, leading to some intracellular accumulation of oxidized thioredoxin and the subsequent decrease in its activity. Despite this fact, under the oxidative stress conditions tested here, Trx activity of *L. ferriphilum* always remained above baseline level observed in the control conditions, suggesting that thioredoxin system could play a pivotal role in defense against oxidative growth conditions. In addition, in this bacterium Trx1, Trx6 and TR can be considered stress response elements, since their gene expression increased dramatically in the presence of oxidative stress elicitors. We can thus conclude that the intracellular levels of Trx activity depend on a fine balance between the levels of *trx* genes expression, the oxidizing conditions of the cellular environment and the levels of TR activity.

Differences in levels of Trx and TR activity under different oxidative stress conditions raise intriguing question regarding the type of signal and the regulators involved in gene expression control and protein activity modulation in the Leptospirilli. While Trx activity increased upon exposure to ferric iron and diamide, no changes were observed in the presence of H_2_O_2_. However, TR activity resulted to be activated with all three oxidative stress elicitors assayed in this study. Certainly, H_2_O_2_ could have an effect on stability of Trx as has been previously determined in *Rhodobacter sphaeroides* [57]. Nevertheless, differences may also exist between the molecular machineries involved in regulation of gene expression triggered by each elicitor. In many bacteria, the OxyR [58] and PerR [59] regulators control the expression of *trxB,* and other genes involved in basic physiological processes, in response to peroxide stress. In *Leptospirillum* spp., molecular components responsible for *trx* genes expression regulation are still to be defined. However, presence of an ortholog of the peroxide sensitive Per-type regulator in the genomes of sequenced Leptospirilli [60] suggests that this regulator could be involved in transcriptional control of the gene encoding TR and possibly also other *trx* genes. These predictions await experimental validation.

Finally, it should be noted that the role of thioredoxin in the oxidative stress response goes beyond the mere recovery of oxidized proteins. Over the past few years, an increasing number of thiol-containing proteins have been identified that use ROS as a mediator to quickly regulate their protein activity [61]. Upon return to non–oxidative stress conditions, cellular reductants such as thioredoxin reduce the oxidized cysteine/s and restore the original protein activity [1], [62]. Interestingly, thioredoxin system has been shown to be associated with activity modulation of proteins involved in the oxidative stress response, including superoxide dismutase (Sod) from *E. coli* [63] and alky hydroperoxide reductase (AhpC) from *H. pylori* [64]. It is therefore not entirely a surprise that activation of thioredoxin system involves a global activation of the cellular components that participate in oxidative stress response. Thus, thioredoxin–based thiol/disulfide system could play a relevant role in oxidative stress response and survival of *Leptospirillum spp*. in the highly oxidizing conditions imposed by bioleaching environments. Elements involved in ROS scavenging in this group of microorganisms have been predicted, including several peroxidases and peroxiredoxins [38], yet a connection between the activity of these elements and the thioredoxin system has not established.

These results pave the way towards a better understanding of the molecular components involved in antioxidant protection and biomolecules repair in iron oxidizing bacteria inhabiting extreme acidic and metal loaded environments.

## Supporting Information

Figure S1 **Phylogram of predicted thioredoxin from** ***Leptospirillum “*** **5 way CG”.** Deduced amino acid sequences of Trx (Trx1-Trx12) were aligned with proteins of known function belonging to Trx family. The proteins used in this analysis were TrxA, TrxC, TrxG, DsbA, DsbC, DsbD, DsbE, DsbG, CcdA, and peroxiredoxins Prx1 and Prx2. The proteins sequences were obtained from genomic databases of *Escherichia coli (Ec), Bacillus subtilis* (Bs), *Shewanella baltica* (Sb), *Bacteroides fragilis* (Bf) or *Dyadobacter fermentans* (Df). Two uncharacterized thioredoxins (Trx) were obtained from *Methanocaldococcus jannaschii* (Mj) and *Acidithiobacillus ferrooxidans* (Af). The corresponding access codes were given in materials and methods. Phylogram was constructed using Neighbor-Joining Algorithm.
(TIF)
