# Supplementary figures and images for: Thiol/Disulfide System Plays a Crucial Role in Redox Protection in the Acidophilic Iron-Oxidizing Bacterium Leptospirillum ferriphilum
Source: PLoS One. 2012 Sep 6;7(9):e44576. doi: 10.1371/journal.pone.0044576 (PMC3435265; doi:10.1371/journal.pone.0044576)

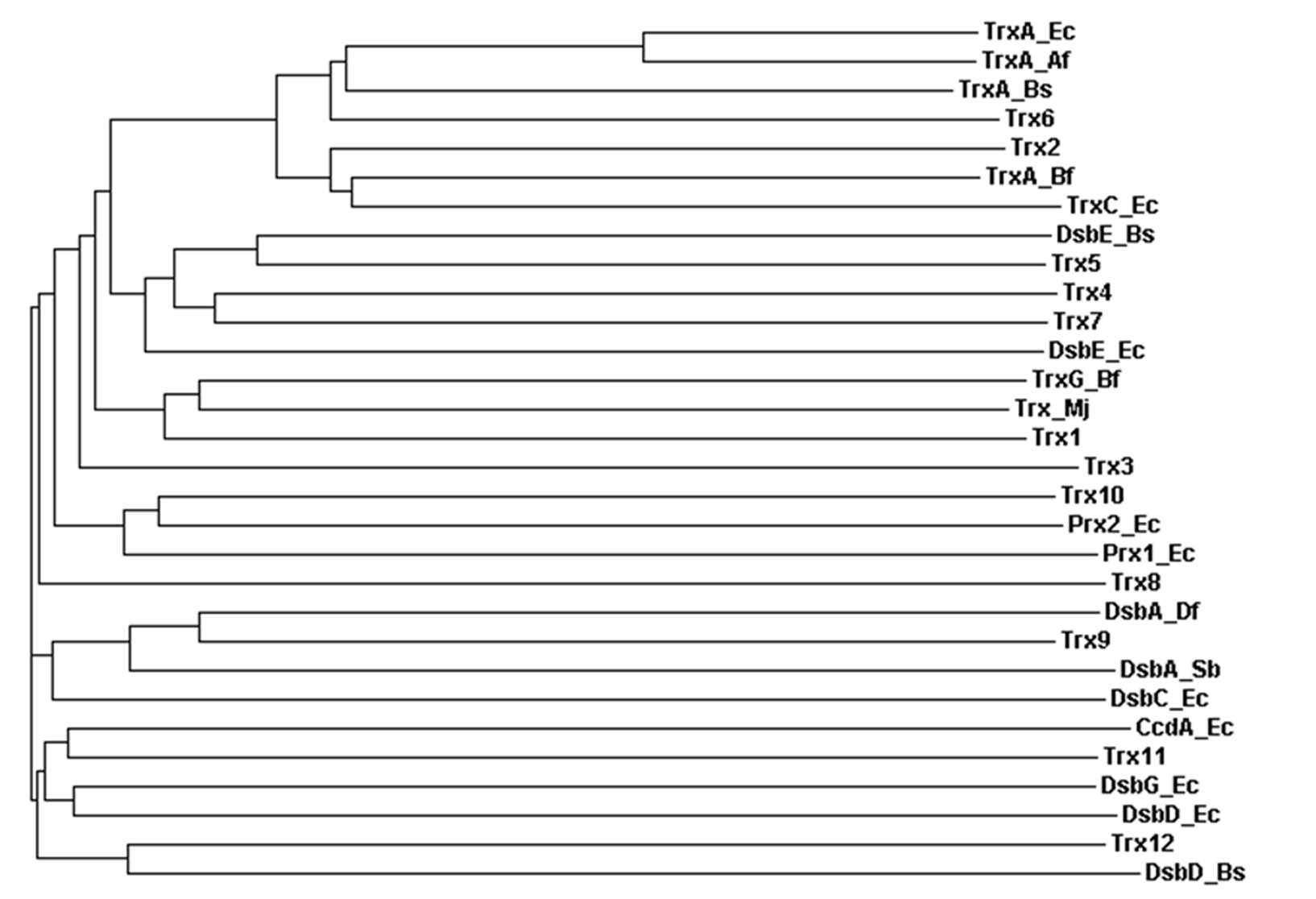

Supplement: Figure S1 — Phylogram of predicted thioredoxin from Leptospirillum “ 5 way CG”. Deduced amino acid sequences of Trx (Trx1-Trx12) were aligned with proteins of known function belonging to Trx family. The proteins used in this analysis were TrxA, TrxC, TrxG, DsbA, DsbC, DsbD, DsbE, DsbG, CcdA, and peroxiredoxins Prx1 and Prx2. The proteins sequences were obtained from genomic databases of Escherichia coli (Ec), Bacillus subtilis (Bs), Shewanella baltica (Sb), Bacteroides fragilis (Bf) or Dyadobacter fermentans (Df). Two uncharacterized thioredoxins (Trx) were obtained from Methanocaldococcus jannaschii (Mj) and Acidithiobacillus ferrooxidans (Af). The corresponding access codes were given in materials and methods. Phylogram was constructed using Neighbor-Joining Algorithm. (TIF) [file pone.0044576.s001.tif]
